# Dietary Restraint Partially Mediates the Relationship between Impulsivity and Binge Eating Only in Lean Individuals: The Importance of Accounting for Body Mass in Studies of Restraint

**DOI:** 10.3389/fpsyg.2016.01499

**Published:** 2016-10-04

**Authors:** Jaime A. Coffino, Natalia C. Orloff, Julia M. Hormes

**Affiliations:** Department of Psychology, University at Albany, State University of New York, Albany, NYUSA

**Keywords:** binge eating, dietary restraint, impulsivity, body mass index, overweight, obesity

## Abstract

Binge eating is characteristic of eating and weight-related disorders such as binge eating disorder, bulimia nervosa, and obesity. In light of data suggest impulsivity is associated with overeating specifically in restrained eaters, this study sought to elucidate the exact nature of the associations between these variables, hypothesizing that the relationship between impulsivity and binge eating is mediated by restrained eating. We further hypothesized that the role of dietary restraint as a mediator would be moderated by body mass index (BMI). Study participants (*n* = 506, 50.6% female) were categorized based on self-reported BMI as under- and normal-weight (BMI < 25, 65.8%, *n* = 333) or overweight and obese (BMI ≥ 25, 34.2%, *n* = 173) and completed the “restrained eating” subscale of the Dutch Eating Behavior Questionnaire, the “impulse control difficulties” subscale of the Difficulties with Emotion Regulation Scale, and the Binge Eating Scale. Findings provide initial evidence for the hypothesized moderated mediation model, with dietary restraint partially mediating the relationship between impulsivity and binge eating severity only in lean respondents. In respondents with overweight or obesity, impulsivity was significantly correlated with binge eating severity, but not with dietary restraint. Findings inform our conceptualization of dietary restraint as a possible risk factor for binge eating and highlight the importance of accounting for body mass in research on the impact of dietary restraint on eating behaviors.

## Introduction

Binge eating refers to the consumption of an objectively larger amount of food than would normally be consumed in one sitting (APA, 2013), and is characteristic of several eating and weight disorders, including bulimia nervosa, binge eating disorder (BED), and obesity (Polivy and Herman, 1985; Stice et al., 2002; Yanovski, 2002; Bas et al., 2008). In addition to the serious adverse health effects of binging, the subjective sense of lack of control over their eating behavior experienced by some binge eaters is a source of significant psychological distress (Didie and Fitzgibbon, 2005; Colles et al., 2008).

Dietary restraint refers to an attempt to restrict food intake for the purpose of weight loss or the prevention of weight gain (Herman and Mack, 1975). Dietary restraint has long been thought of as a predictor of the onset and maintenance of binge eating, particularly in individuals who unsuccessfully attempt to restrict intake (Johnson et al., 2012). Growing evidence suggests an association between dietary restraint and excess weight in diverse populations, including men, women, boys, and girls (Lluch et al., 2000). Early studies also found that restrained eating was positively correlated with binge eating severity in respondents with obesity (Marcus et al., 1985). However, more recent longitudinal work has not been able to replicate the finding of a significant association between dietary restraint and binge eating (Spoor et al., 2006). There are several possible explanations for these discrepancies in the literature, including a failure to account for mediating and moderating variables in the relationship between dietary restraint and binge eating.

### The Role of Impulsivity

Impulse control difficulties and binge eating are linked, such that higher levels of impulsivity are associated with greater binge frequency (Jansen et al., 2009). Impulsive behavior is found in individuals who lack foresight and act in a spontaneous manner, and recent research found impulsivity to be common among people who overeat and experience undesirable weight gain (Davis, 2009).

Dawe and Loxton (2004) describe impulsivity as having two main components, namely (1) reward/sensitivity drive and (2) rash, spontaneous impulsiveness. Reward/sensitivity drive occurs when individuals are more driven by immediate versus delayed rewards (Dawe and Loxton, 2004), and may contribute to the decision to engage in a binge eating episode in order to experience the immediate satisfaction associated with the consumption of a craved food (as opposed to other, more distant or uncertain rewards, such as weight loss). Rash-spontaneous impulsiveness occurs when individuals suffer from disinhibited responses, including loss of control over their eating behaviors, which can result in the compulsive overconsumption of food. Studies found that individuals with BED have higher food-related reward sensitivity and rash-spontaneous impulsiveness when compared to controls (Schag et al., 2013).

### The Importance of Body Mass

Research has thus established a link between restraint and binge eating, as well as between impulse control and binge eating, though the exact nature of the relationships between these three factors remains to be elucidated further, especially in light of inconsistent findings from prior studies. For example, a 1994 study failed to find a significant association between impulsivity ratings and frequency of binge eating episodes in bulimic respondents (Wolfe et al., 1994). Nederkoorn et al. (2004) report findings of an association between dietary restraint and impulsivity, such that restrained eaters displayed an inhibitory control deficit when compared to unrestrained eaters. Specifically, restrained eaters had a difficult time inhibiting their ongoing responses during a stop-signal task (Nederkoorn et al., 2004). This is in contrast to earlier work that found urge to binge and dietary restraint to be unrelated in impulsive individuals (Steiger et al., 1999), suggesting that an underlying factor other than dietary restraint may account for the urge to binge in impulsive individuals. Other studies found that impulsivity is associated with binge eating specifically in restrained eaters (Jansen et al., 2009), suggesting that restrained eating alone may not be causing binge eating, but could matter specifically in those also engaged in impulsive overeating.

Much of the research on binge eating, including studies that link dietary restraint to binging severity, has focused specifically on overweight or obese populations (Marcus et al., 1985). The small body of research that has been conducted on normal weight individuals suggests that the mechanisms underlying binge eating behavior may differ in important ways depending on body mass. For example, in a study that examined primarily normal weight individuals, binge eating was linked to dietary control, although it was not associated with impulsivity (Steiger et al., 1999). Specifically, in participants with low to moderate impulsivity, dietary restraint was correlated with urge to binge. However, in highly impulsive participants, there was no relationship between dietary restraint and urge to binge. It is important to note that although this sample consisted of normal weight individuals, participants had prominent bulimic symptoms.

In a study that examined normal weight healthy women, self-reported impulsiveness and behavioral impulsivity were significant predicators of consumption, but dietary restraint was not a significant predictor of food intake (Guerrieri et al., 2007). However, in this sample, the lack of association between dietary restraint and food intake may have been due to overall moderate scores on the Restraint Scale. Further research in individuals of diverse weights is thus needed to systematically examine how the relationships between impulsivity, dietary restraint, and binge eating may differ depending on body mass.

### Aims and Hypotheses

**FIGURE 1:**
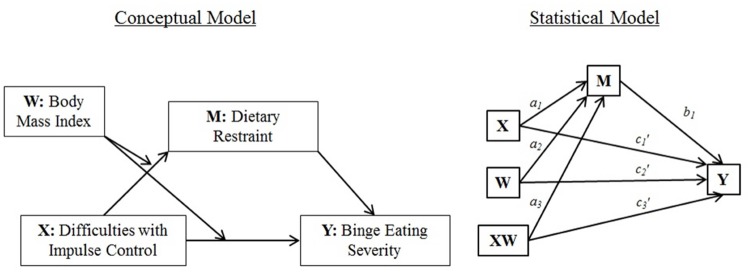
**Conceptual and statistical moderated mediation model [adapted from (Hayes, 2012)]**.

In light of conflicting data from prior research, the present study sought to further elucidate the nature of the relationships between impulsivity, dietary restraint, and binge eating. We hypothesized that the relationship between impulsivity and binge eating is mediated by restrained eating, but that body mass (i.e., under- and normal-weight versus overweight and obese) moderates the role of restraint as a mediator (see **Figure 1** for hypothesized moderated mediation model).

## Materials and Methods

All methods were reviewed and approved by the local Institutional Review Board. Study participants were informed of the nature and purpose of the research and consented prior to completion of questionnaires. All procedures followed were in accordance with the Declaration of Helsinki.

### Participants

Undergraduate students (*n* = 610; see **Table 1** for demographics) at a large university in the northeastern region of the United States came into the laboratory in groups of up to 15 at a time to complete an online survey hosted on the secure server SurveyMonkey while seated at individual computer stations to ensure privacy. Response rate was 100%. Participants were recruited via the psychology department’s research pool website and received research participation credit in exchange for time spent in the laboratory.

**Table 1:** Demographics, body mass index, and mean scores on the difficulties in Emotion Regulation Scale, Dutch Eating Behavior Questionnaire, and the Binge Eating Scale in under- and normal-weight versus overweight and obese respondents.

	Total	Under/Normal	Over/Obese	Statistics
	(*n* = 506)	(*n* = 333)	(*n* = 173)	
	*M* (*SD*)/% (*n*)	*M* (*SD*)/% (*n*)	*M* (*SD*)/% (*n*)	
**Demographics**				
Age	18.84 (1.76)	18.75 (1.59)	19.00 (2.04)	*t*(504) = -1.52, *p* = 0.13, *d* = -0.14
% Non-white	43.5 (220)	43.8 (146)	42.8 (74)	X^2^= 0.05, *p* = 0.85, φ = 0.01
% Female	50.6 (256)	53.5 (178)	45.1 (78)	X^2^= 3.19, *p* = 0.08, φ = 0.08
Body Mass Index	23.84 (3.95)	21.64 (1.99)	28.08 (3.28)	*t*(504) = -23.66, *p <* 0.001, *d* = -2.37
**Difficulties in Emotion Regulation Scale**				
Impulse Control Difficulties	2.12 (0.80)	2.07 (0.76)	2.20 (0.86)	*F*(1,464) = 2.81, *p* = 0.09, $\eta_{p}^{2}$ = 0.01
**Dutch Eating Behavior Questionnaire**				
Restrained	2.43 (0.91)	2.23 (0.88)	2.81 (0.85)	*F*(1,455) = 45.36, *p <* 0.001, $\eta_{p}^{2}$ = 0.09^a^
**Binge Eating Scale**				
Total Score	10.54 (7.59)	9.43 (7.09)	12.69 (8.06)	*F*(1,444) = 27.09, *p* < 0.001, $\eta_{p}^{2}$ = 0.06^a^

*^a^Gender included as significant covariate (*p* < 0.05).*

### Measures

Participants indicated basic demographics, including gender, age, and race/ethnicity. Self-reported height and weight were used to calculate body mass index (BMI). Participants then completed the following widely used and well-validated measures. Of note, the questionnaire included additional measures of eating behaviors, but those responses were not analyzed for the purposes of the present study.

#### Difficulties in Emotion Regulation Scale (DERS) – “Impulse Control Difficulties” Subscale

The DERS is a 36-item questionnaire that assesses six areas of emotion regulation difficulties, including “nonacceptance of emotional responses,” “difficulties engaging in goal-directed behavior,” “difficulties with impulse control,” “lack of emotional awareness,” “limited access to emotion regulation strategies,” and “lack of emotional clarity” (Gratz and Roemer, 2004). All items are rated on a 5-point Likert scale ranging from “almost never” to “almost always.”

For the purpose of this study, we only focused on ratings of “impulse control difficulties” a subscale of the DERS that includes statements such as “When I’m upset, I feel out of control” and “When I’m upset, I feel like I can remain in control of my behaviors.” The Barratt Impulsiveness Scale is often used in studies examining impulsivity, however, the DERS is a psychometrically sound alternative that more specifically quantifies the concept of losing control over behaviors (Patton and Stanford, 1995).^1^ Given that binge eaters often report feeling a loss of control over their eating behaviors, the DERS is thus more suitable for the present investigation. Of note, previous studies of binge eating have also utilized the “impulse control difficulties” subscale of the DERS (Whiteside et al., 2007). In the present sample, the Cronbach α coefficient for the subscale was 0.82 for the under- and normal-weight participants and 0.85 for the overweight and obese participants.

#### Dutch Eating Behavior Questionnaire (DEBQ) – “Restrained Eating” Subscale

The DEBQ is a 33-item questionnaire that measures tendencies to eat in response to “emotional” and “external” cues, as well as “restrained eating” (van Strien et al., 1986). The DEBQ “restrained eating” subscale measures dietary restriction (e.g., “Do you try to eat less at mealtimes than you would like to eat?”) and is widely accepted as accurately measuring the restriction of calories in eating behaviors that occur in everyday life (Laessle et al., 1989). In comparisons of the DEBQ to other commonly used measures of dietary restraint, including the Revised Restraint Scale (Herman and Polivy, 1980; Polivy et al., 1988) and the Three Factor Eating Questionnaire (Stunkard and Messick, 1985; Cappelleri et al., 2009), the DEBQ proved to be psychometrically sound with high test–retest reliability, high internal consistency, and highly stable factor structure across genders, weight categories, and random samples (Allison et al., 1992). In addition, the DEBQ has been cited as being easier to complete because it does not ask open-ended questions about body size or weight fluctuation (Heatherton et al., 1988). Only scores on the “restrained eating” subscale were included in the present analyses. In this sample, the Cronbach’s α coefficient for the “restrained eating” subscale of the DEBQ was 0.93 for the under- and normal-weight participants and 0.91 for the overweight and obese participants, suggesting excellent internal consistency.

#### Binge Eating Scale (BES)

The BES is a 16-item questionnaire assessing binge eating severity. The BES was originally validated in an obese population (Gormally et al., 1982). The questionnaire quantifies both behavioral outcomes (e.g., “I have a strong habit of eating when I’m bored. Nothing seems to help me break the habit.”) and cognitions associated with binge eating (e.g., “Almost all the time I experience strong guilt or self-hate after I overeat.”). Items are scored on a scale ranging from 0 to 2 or 3, resulting in a possible total score ranging from 0 to 46, with higher scores indicating greater binge eating severity. A score of ≤17 indicates “mild or no binge eating,” a score of 18–26 suggests the presence of “moderate binge eating,” and a score of ≥27 indicates “severe binge eating” (Gormally et al., 1982). For this sample, the Cronbach α coefficient was 0.88 for the under- and normal-weight participants and 0.89 for the overweight and obese participants.

### Statistical Analyses

Statistical analyses were conducted using SPSS version 23 and the PROCESS macro for SPSS (Hayes, 2013). Participants under the age of 18 and those not indicating their gender were excluded from the analyses reported here. Given the focus of the present investigation on differences in eating behaviors by body mass we also excluded responses from participants not indicating either height or weight information, resulting in a final sample of 506 respondents (50.6%, *n* = 256 female, *M*_age_ = 18.84, *SD* = 1.76, range: 18–39). Participants were divided into two groups, combining underweight and normal weight participants (65.8%, *n* = 333) and overweight and obese participants (34.2%, *n* = 173) to facilitate comparisons by weight status.

Under- and normal-weight respondents were compared to individuals with overweight and obesity using chi-square and *t*-tests, as well as univariate and multivariate (for measures containing multiple subscales) analyses of covariance. The hypothesized moderated mediation model was examined using PROCESS (“Model 8”) (Hayes, 2013). All indirect effects were subjected to follow-up bootstrap analyses with 10,000 bootstrap samples and 95% bias corrected confidence intervals (CI). Gender was initially included as a covariate in all analyses but subsequently removed if found not to be significant. The hypothesized role of dietary restraint as a mediator in the relationship between impulsivity and binge eating in under- and normal-weight versus overweight/obese individuals was further examined using Pearson’s product moment coefficients r, linear regression analyses, and PROCESS mediation modeling (“Model 4”) (Hayes, 2013).

## Results

The mean BMI in the group of under- and normal-weight participants was 21.64 (*SD* = 1.99), with 8.1% (*n* = 27) meeting criteria for underweight (i.e., BMI < 18.5) and 91.9% (*n* = 306) being categorized as normal weight (i.e., BMI 18.5–24.9). In under- and normal-weight participants, BMI was significantly and positively correlated with dietary restraint (*r* = 0.28, *p* < 0.001) and binge eating severity (*r* = 0.20, *p* = 0.001), but not with impulse control difficulties (*r* = 0.08, *p* = 0.17). The mean BMI in respondents with overweight and obesity was 28.08 (*SD* = 3.28), with 79.8% (*n* = 138) of respondents falling in the overweight category (i.e., BMI 25.0–29.9) and 20.2% (*n* = 35) meeting criteria for obesity (i.e., BMI ≥ 30.0). In the overweight and obese sample, BMI was significantly correlated with binge eating severity (*r* = 0.26, *p* = 0.001), but not with impulse control difficulties (*r* = 0.06, *p* = 0.46) or with dietary restraint (*r* = 0.14, *p* = 0.09).

Under- and normal-weight respondents differed significantly from participants with overweight and obesity in reported binge eating severity [χ^2^ = 9.84, *p* = 0.01, φ = 0.15]. A majority of under- and normal-weight respondents reported “mild” or “no binge eating” (85.4%, *n* = 247), 13.9% (*n* = 41) endorsed “moderate” binge eating, and 2.0% (*n* = 6) experienced “severe” binge eating. In the overweight and obese sample 71.9% (*n* = 110) reported “mild” or “no binge eating,” 22.9% (*n* = 35) endorsed “moderate” binge eating, and 5.2% (*n* = 8) experienced “severe” binge eating. There was a significant univariate main effect of body mass on total BES scores [*F*(1,444) = 27.09, *p* < 0.001, $\eta_{p}^{2}$ = 0.06, with gender as a significant covariate *p* < 0.001], with significantly higher scores in the overweight and obese sample, compared to those who were either underweight or normal weight (see **Table 1** for descriptives).

Individuals meeting criteria for overweight or obesity scored significantly higher on the “restrained eating” subscale of the DEBQ, compared to under- and normal-weight respondents (see **Table 1** for descriptives and between-subject comparisons). There were no significant between-group differences in scores on the “impulse control difficulties” subscale of the DERS (see **Table 1** for descriptives).

The extent to which the indirect effect of impulse control difficulties (*X*; see **Figure 1** for representations of the conceptual and statistical models tested) on binge eating severity (*Y*) through the hypothesized mediator dietary restraint (*M*) depends on body mass (the hypothesized moderator *W*) was examined using PROCESS “Model 8.” There was no initial support for moderation of the direct effect of impulse control difficulties on binge eating severity by body mass (*c*_3_′ = 0.11, *p* = 0.91; **Figure 1**). Similarly, there was no initial evidence of moderation of the indirect effect (i.e., through dietary restraint) by body mass, as indicated by a lack of a statistically significant interaction between impulse control difficulties and body mass (*a*_3_ = -0.17, *p* = 0.11; **Figure 1**). However, as noted by Hayes (2012), in testing moderated mediation, emphasis should not simply be placed on the significance of the indirect effect of the product *XW* on *Y* through *M*, but instead, moderation of both the direct and indirect effects can be probed by estimating the conditional direct and indirect effects of *X* on *Y* (through *M*) at various levels of the hypothesized moderator *W*. Doing so yielded evidence for a significant positive conditional direct effect of impulse control difficulties on binge eating severity in both under-/normal-weight respondents (2.83, *p* < 0.001) and respondents with overweight and obesity (2.94, *p* < 0.001). Analyses also yielded evidence for a significant positive conditional indirect effect of impulse control difficulties on binge eating severity via dietary restraint in under-/normal-weight respondents (0.66, 95% CI: 0.28, 1.13), but not in overweight/obese individuals (0.18, 95% CI: -0.21, 0.65).

In the under- and normal-weight participants, impulsivity, dietary restraint, and binge eating severity were significantly and positively correlated (see **Table 2** for correlation coefficients). The regression of impulsivity on binge eating, ignoring the hypothesized mediator dietary restraint, was significant [*b* = 3.23, *t*(251) = 5.78, *p* < 0.001]. The regression of impulsivity on dietary restraint was also significant [*b* = 0.21, *t*(251) = 2.87, *p* = 0.004]. The regression of the hypothesized mediator (dietary restraint) onto binging severity, controlling for impulsivity, was also significant [*b* = 3.26, *t*(250) = 7.64, *p* < 0.001]. Finally, when controlling for dietary restraint, impulsivity was still a significant predictor of binge eating [*b* = 2.53, *t*(250) = 4.94 *p* < 0.001]. A Sobel test was conducted and was statistically significant [*z* = 2.67, *p* = 0.01], confirming that dietary restraint partially mediated the relationship between impulsivity and binge eating severity in the under- and normal-weight individuals.

**Table 2:** Correlations between impulse control difficulties, restrained eating, and binge eating severity in under- and normal-weight respondents.

	DEBQ Restrained	BES
	Eating	*r*(*p*)
	*r* (*p*)	
DERS Impulse Control Difficulties	0.16 (0.01)	0.34 (<0.001)
DEBQ Restrained Eating	–	0.45 (<0.001)

In the overweight and obese participants, impulsivity and binge eating were significantly and positively correlated, as were binge eating and restraint, however, impulsivity and dietary restraint were not significantly correlated (see **Table 3** for correlation coefficients). Given initial results from the test of moderated mediation and the lack of a significant association between the hypothesized causal variable (i.e., impulsivity) and the hypothesized mediator (i.e., dietary restraint), follow-up mediation analysis was not carried out in respondents with overweight and obesity.

**Table 3:** Correlations between impulse control difficulties, restrained eating, and binge eating severity in respondents with overweight and obesity.

	DEBQ Restrained	BES
	Eating	*r*(*p*)
	*r* (*p*)	
DERS Impulse Control Difficulties	0.07 (0.37)	0.34 (<0.001)
DEBQ Restrained Eating	–	0.35 (<0.001)

## Discussion

Findings provide initial support for the hypothesis that the relationships between impulsivity, dietary restraint, and binge eating severity are moderated by body weight. Tests of moderated mediation yielded evidence for a significant conditional indirect effect of impulse control difficulties on binge eating severity via dietary restraint only in lean individuals. *Post hoc* analyses confirmed that in the under- and normal-weight sample, dietary restraint partially mediated the relationship between impulsivity and binge eating severity. This suggests that dietary restraint serves as a proximal predictor of binge eating in normal weight, impulsive individuals. Given that respondents were of healthy weight, findings are somewhat inconsistent with the widespread perception that dietary restraint is ultimately an ineffective strategy for weight loss and maintenance, and instead provide preliminary support for an alternative conceptualizations of dietary restraint as an effective attempt at self-control around food that may be beneficial in maintaining a healthy BMI (Johnson et al., 2012). However, studies have also shown that even though dietary restraint may initially control eating behaviors, it is ultimately unsuccessful as a long-term means of dietary control (Lowe and Timko, 2004). More research is therefore needed to determine if normal weight individuals engaged in dietary restraint to manage urges to binge are successful in the long run, or ultimately end up at risk for overweight and obesity.

In the overweight and obese sample, impulsivity exerted a statistically significant conditional direct effect on binge eating severity, but there was no evidence to suggest that this relationship was mediated by dietary restraint. This finding suggests that impulsivity is a more proximal antecedent to binge episodes in overweight and obese individuals, who do not appear to engage in dietary restraint in an attempt to manage urges to engage in binge eating episodes. These important differences between lean and overweight/obese respondents in proximal risk factors for binge eating may in part account for some of the discrepancies in prior research on the relationships between impulsivity, dietary restraint, and binge eating severity.

Findings thus highlight the need to account for body weight in studies of dietary restraint and the mechanisms underlying binge eating. They also have potentially important implications for prevention and treatment interventions. Binge eating may be a risk factor for, or symptom of more serious eating pathology, such as BED or bulimia nervosa (APA, 2013). Furthermore, the loss of control associated with binge eating episodes is often perceived as distressing by patients (Colles et al., 2008). Given the importance of targeting proximal predictors of binge eating during treatment, dietary restraint may be an appropriate risk factor to target specifically in lean patients endorsing binging behavior. In overweight and obese binge eaters, dietary restraint was not associated with binge eating. Instead, impulsivity emerged as a direct predictor of binge eating severity, suggesting that strategies for improved impulse control should be taken into consideration when developing treatment interventions targeting binge eating specifically in individuals with overweight and obesity. Prior research suggests that impulsivity affects obesity risk even at a young age, when the most impulsive children are also the ones who are the most obese (Nederkoorn et al., 2007). It may therefore be important to target impulse behavior at a young age before eating pathology becomes an issue.

There are several limitations to the present research that must be noted. Measures were completed by psychology undergraduate students. Relatively few respondents at all levels of BMI endorsed moderate or severe levels of binge eating. Only about a third of the current sample met criteria for categorization as “overweight/obese.” Comorbid conditions such as Impulse Control Disorders or Attention Deficit and Hyperactivity Disorder were not assessed. This may limit the extent to which findings generalize to more demographically diverse populations. Future work should seek to assess the extent to which findings may translate to more diverse populations, including clinical samples of patients of diverse body mass with diagnosed eating disorder pathologies characterized by the presence of binge eating episodes. All data were based on self-report and future research should seek to replicate findings using more objective measures, including behavioral measures of impulsivity. The cross-sectional nature of the data prevented us from assessing the direction of causality in the relationship between impulsivity, dietary restraint, and binge eating severity. It has been suggested that dietary restraint is a strategy employed by some in response to weight gain, as opposed to being an antecedent of accumulation of excess body fat (Johnson et al., 2012). More research is needed to examine this possibility.

## Conclusion

It has been well established in the research literature that individuals who engage in binge eating are more likely to be overweight and obese. Obesity is a major public health concern as the number of obese individuals within the United States continues to increase. Obesity is also of concern because it contributes to and exacerbates many other physical and mental health problems. It is important to identify the underlying mechanisms that cause binge eating in order to begin to address this trend.

Lack of impulse control was a common risk factor for binge eating in both normal weight and overweight binge eaters, whereas dietary restraint emerged as a proximal predictor of binging severity only in lean respondents. Current research on binge eating primarily focuses on individuals with overweight and obesity. Future research should include both participants of diverse weights to further elucidate differences by body mass. Differences in risk factors for binge eating by weight status may inform the development of prevention and treatment interventions, as well as our understanding of other forms of eating pathology.

## Author Contributions

NO and JH designed the study and collected the data on which the present analyses are based. JC, NO, and JH developed the study aims and hypotheses and conducted the statistical analyses. All authors were involved in the writing of the manuscript and approve of the draft in its current form.

## Conflict of Interest Statement

The authors declare that the research was conducted in the absence of any commercial or financial relationships that could be construed as a potential conflict of interest.

## Footnotes

1 Given that the convergent validity of the DERS “impulse control difficulties” subscale with other established measures of impulsivity has not yet been systematically evaluated, it should be noted that participants in the present study also completed the 30-item Barratt Impulsiveness Scale (BIS-11), a well validated and psychometrically sound measure of “attentional” (Cronbach’s α in the present sample = 0.67; *M* = 2.14, *SD* = 0.47), “motor” (Cronbach’s α = 0.70; *M* = 2.04, *SD* = 0.41) and “non-planning” impulsivity (Cronbach’s α = 0.72; *M* = 2.31, *SD* = 0.45) (Barratt, 1959; Stanford et al., 2009). The DERS “impulse control difficulties” subscale correlated positively and significantly with all three BIS-30 subscales (*r* = 0.36, *p* < 0.011, *r* = 0.23, *p* < 0.011, and *r* = 0.36, *p* < 0.001, respectively), supporting its use in the present investigation to quantify impulsivity as it relates to loss of control over eating behaviors.
